# THE EFFECTS OF CAREGIVING HISTORY ON THE WELL-BEING OF INFORMAL CAREGIVERS LATER IN LIFE

**DOI:** 10.1093/geroni/igae098.1513

**Published:** 2024-12-31

**Authors:** Sarah Neller, Radion Svynarenko

**Affiliations:** University of Tennessee Knoxville, Knoxville, Tennessee, United States; University of Tennessee, Knoxville, Tennessee, United States

## Abstract

Previous studies have been primarily focused on documenting the negative impact of caregiving on quality of life. However, such a perspective overlooks the potentially positive outcomes that it can bring into the caregiver’s life. The purpose of the current examination was to explore how the history of informal caregiving for six months or more influences beliefs about well-being later in life. We used data from the Health and Retirement Study on caregiving history from the Life History Surveys, which were combined with well-being measures from Psychosocial and Lifestyle Questionnaires collected between 2016 and 2022. For data analysis, we used a set of Ordinary Least Squares regression models (OLS) to control for age, gender, race, ethnicity, education, and marital status. The total sample size in each OLS model varied depending on the year of data collection and response rates for each dependent variable. We found that history of caregiving has mixed effects on well-being later in life. Though it was negatively associated with life satisfaction (b = -0.19, p < 0.001, n = 3,902), it was positively associated with compassionate goals (b = 0.094, p< 0.001, n = 3,860) and purpose in life (b = 0.07, p = 0.017, n = 4,713), and negatively associated with fears about aging (b = -0.07, p = 0.016, n = 3,161). We will discuss potential ways to leverage positive wellbeing effects to promote caregivers’ flourishing and positive caregiver outcomes. These findings can be used to develop future interventions that aim to improve well-being of informal caregivers.

